# Validation of the Spanish version of the Goodman score in total hip arthroplasty

**DOI:** 10.1186/s13018-021-02653-6

**Published:** 2021-08-20

**Authors:** Julián Brañes, Maximiliano Barahona, Sebastián Carvajal, Rodrigo Wulf, Cristián Barrientos

**Affiliations:** 1 Orthopaedic Department, Hospital Clinico San José, 999 Santos Dumont Street, 3rd Floor, Office 351, 8380456 Santiago, Independencia Chile; 2 Orthopaedic Department, Hospital Clinico Universidad de Chile, Santiago, Chile; 3 Orthopaedic Department, Clinica Santa María, Santiago, Chile

**Keywords:** Total hip arthroplasty, PROMs, Cross-cultural adaptation, Translation, Satisfaction, Validation studies

## Abstract

**Purpose:**

Currently, patient-reported outcome measures (PROMs) are the standard instruments used to compare arthroplasty results. Goodman et al. recently published a well-constructed scale with excellent psychometric properties that can be quickly administered. The main objective of our study was to translate, culturally adapt, and validate a Spanish version of the Goodman questionnaire in patients who underwent total hip arthroplasty (THA).

**Methods:**

The original Goodman scale was translated into Spanish and cross-culturally adapted. Then, the data from this version were tested for psychometric quality. We designed a cross-sectional study for data collection. This study enrolled 2 institutions. Patients who underwent hip replacement due to primary osteoarthritis secondary to dysplasia between 1 January 2018 and 31 December 2019 were included. A total of 153 patients were contacted twice to record the Goodman and Oxford hip scales (OHS) to assess the validity of the questionnaire. Reliability was tested using the Cronbach’s alpha, Concordance using 3 test: intraclass correlation coefficient (ICC), Lin's concordance correlation coefficient (CCC), and the Bradley-Blackwood *F* test. The spearman correlation was used to asses correlation between the OHS and the Spanish-adapted Goodman scale.

**Results:**

The overall satisfaction after THA was reported to be “very satisfied” by 137 patients (75%), and only 14 patients reported some degree of dissatisfaction (6%). The improvement in quality of life was reported to be “more than I ever dreamed possible” by 41% patients. Cronbach’s alpha was acceptable, reaching a coefficient of 0.95 (95% confidence interval, 0.82–1). No statistical difference (*t* test, *p* = 0.55) was found in the original version, with great internal validity. Test re-test concordance was optimal among the 3 tests used. A moderate correlation was found between the OHS and the Spanish-adapted Goodman scale.

**Conclusion:**

The Spanish version of the Goodman questionnaire in THA is a reliable, consistent, and feasible scale to evaluate patient satisfaction and improvement in the quality of life in Spanish speakers.

**Supplementary Information:**

The online version contains supplementary material available at 10.1186/s13018-021-02653-6.

## Introduction

Total hip arthroplasty (THA) is the gold standard in the treatment of severe hip osteoarthritis with intractable pain. THA improves the quality of life to such a degree that it was labeled as the surgery of the twentieth century [1].

Nevertheless, there is still room for further improvement. It is essential to develop instruments to compare results between different surgical approaches, prosthetic models, type of hospitalization, and pain management, to name a few [2, 3]. The current focus is being placed on patient-reported outcome measures (PROMs), which is the standard to make these comparisons. A perfect PROM has to be reliable, accessible, validated, responsive to change, and measure clinical results with minimal administrative burden [4]. The use of PROMS in the literature has markedly increased in the past decade, with the Harris hip score being the most frequently used in THA [5]. Different countries have developed national registries using PROMS to evaluate their outcomes, with language and cultural interpretations being limitations to their use and extrapolating the results [6, 7]

Goodman et al. [8] recently published a well-constructed scale with excellent psychometric properties that can be quickly administered–only 5 questions–and that allows estimation of patient satisfaction with THA. Although the score achieves a moderate correlation with pain and function, it has different dimensions; therefore, it should be evaluated independently. We believe that assessing patient satisfaction is crucial in THA; however, this scale is not in Spanish. According to the data of 2020, about 489 million people were native Spanish speakers, and 585 million (7.5% of the world population) speak Spanish [9]. Hence, it is of immense value to translate and perform a cultural adaptation of the new assessment tools to Spanish to extend their application.

The main objective of our study was to translate, culturally adapt, and validate a Spanish version of the Goodman questionnaire in patients who underwent total hip arthroplasty. We hypothesize that systematic translation and adaptation will lead to the development of a questionnaire with equal reliability and consistency as that of the English version.

## Methods

This study was designed in 2 phases. First, the original Goodman scale was translated into Spanish and cross-culturally adapted. Following the implementation of the Spanish version, the psychometric quality of the data was tested. This study was approved by the ethics review board.

The Goodman satisfaction scale consists of 2 items: the first assesses satisfaction through 4 questions, each rated on a 5-point Likert scale. The satisfaction summary score corresponds to the non-weighted average of the 4 questions, which correspond to 100, 75, 50, 25, and 0 according to the nominal response (for example, “very satisfied” corresponds to score 100 and “very dissatisfied” to 0). The second item aimed to evaluate the quality of life using a single question. This scale is valid and reliable, with high internal consistency and feasibility, and can be used postoperatively to assess the impact of THA [7].

The translation and cross-cultural adaptations were performed according to the recommended methodology through the following step s[10]: first, a forward translation to Spanish was performed by 2 translators whose mother tongue was Spanish and fluent in English. A back-translation to English was carried out by 2 translators whose mother tongue was English and fluent in Spanish. A consensus meeting including 3 experts in THA, translators, and medical students compared the original English version and the back-translated version for consistency. In the same session, the clinical-linguistic issues in the Spanish-translated version were addressed. Then, the final version was tested for the level of understanding on 30 randomly selected patients who underwent THA.

We designed a cross-sectional study for data collection, and 2 institutions were enrolled: a university and a public hospital. Both centers had access to the same type and brands of prostheses to perform surgery, and surgeons had similar levels of training; 2 of the surgeons work in both centers, and the orthopaedic departments of both centers conducted joint academic activities regularly and shared a training program for fellow hip surgeons.

Patients were included if they were above 18 years of age and underwent hip replacement due to primary osteoarthritis secondary to dysplasia between 1 January 2018 and 31 December 2019. In all cases, the posterior hip approach and a primary non-constrained prosthesis were used (Corail® uncemented hip stem, Pinnacle® uncemented cup: DePuy Synthes, Warsaw, IN). All hip fractures, hip revisions, infections, and bilateral cases were excluded, as were patients with language or mental impairment.

A total of 520 patients were eligible for assessment. The medical records were screened to collect sociodemographic and clinical data, including sex, age, date of surgery, surgery protocol, phone, and e-mail.

**Fig. 1:**
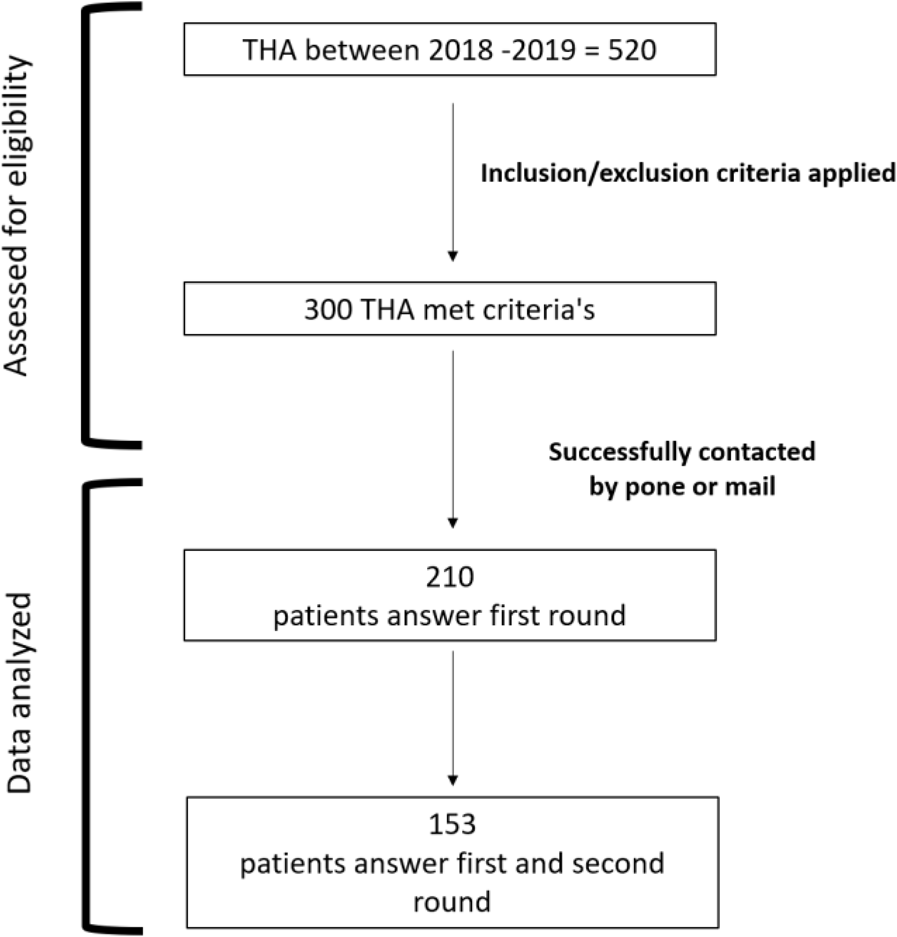
Flow chart depicting study methodology

A total of 300 patients met the selection criteria, and attempts to contact them were initiated in June 2020; therefore, all patients had at least 1 year of follow-up after THA. Of them, 210 patients were successfully contacted by phone or mail, provided digital written or verbal informed consent to be enrolled in the study, and responded to the Goodman scale and the Oxford hip scale (OHS). The OHS is a globally accepted scale which was validated in Spanish [11], consisting of 12 questions to evaluate patients’ functionality, and ranges from 12 to 60. The lower the score, the better the outcome. The OHS was used to assess the validity of the adapted Goodman scale [12]. The scale was sent again 14 days after the first response was received. A total of 153 patients returned the second round of answers for analysis (Fig. 1).

Cronbach's alpha was estimated for reliability (internal consistency) analysis. A coefficient greater than 0.75, was considered acceptable [13]. The alpha coefficient was assumed to be normally distributed, and *t* test was used to compare the Cronbach's alpha between this study and that performed by Goodman et al. [8]. In addition, factor analysis was performed, and the factors were maintained if the eigenvalue was above 1.

The test-retest was assessed by comparing the first response with the answers obtained 14 days later. The intraclass correlation coefficient (ICC), Lin's concordance correlation coefficient (CCC), and the Bradley-Blackwood F test were used. An ICC and CCC over 0.75, were considered acceptable; meanwhile, a probability over 0.15, was deemed acceptable to validate the null hypothesis of concordance in the Bradley-Blackwood *F* test [14].

Finally, Spearman correlation (rho) test was conducted using the OHS score to validate the Spanish-adapted Goodman scale. An absolute rho above 0.6, was interpreted as good correlation [15].

## Results

A total of 210 patients answer the first round of questions. The median age was 68 years old (range, 23 to 89; interquartile range, 59 to 74), and 136 patients were female (64.76%). The median score of OHS and the Spanish-adapted Goodman scale is described in Table 1.

**Table 1:** Satisfaction summary score of the Spanish adaptation of the Goodman scale and the Oxford hip scale in rounds 1 and 2

	S-A Goodman satisfaction	OHS
Round 1 (*n*)	210	210
- Median (range)	100 (0 to 100)	19 (12 to 56)
- IQR	81.3 to 100	15 to 33
Round 2 (*n*)	153	153
- Median (range)	100 (0 to 100)	19 (12 to 53)
-IQR	87.5 to 100	15 to 28

Abbreviations: *S-A* Spanish-adapted, *IQR* interquartile range

The overall satisfaction after THA was reported to be "very satisfied" by 137 patients (75%), and only 14 patients reported some degree of dissatisfaction (6%) (Table 1). Table 1 shows the satisfaction summary score of the Spanish-adapted Goodman and the OHS scores obtained in both rounds. The improvement in quality of life was reported to be “more than I ever dreamed possible” by 81 patients (41%), “great improvement” by 89 patients (42%), “moderate improvement” by 25 (12%), “a little improvement” by 7 patients (3%), “no improvement” by 2 patients (1%) and “worse quality of life” by only 6 patients (3%) (Table 2).

**Table 2:** Summary of questions on patient satisfaction dimension of the Spanish adaptation of the Goodman scale in round 1

Satisfaction	Pain relief	Housework or yard work	Recreational activities	Overall
Very satisfied	**160 (76%)**	**136 (65%)**	**124 (59%)**	**157 (75%)**
Somewhat satisfied	**30 (14%)**	**48 (23%)**	**53 (25%)**	**31 (15%)**
Neither satisfied nor dissatisfied	**5 (2%)**	**9 (4%)**	**17 (8%)**	**8 (4%)**
Somewhat dissatisfied	**10 (5%)**	**7 (3%)**	**8 (4%)**	**8 (4%)**
Very dissatisfied	**5 (2%)**	**10 (5%)**	**8 (4%)**	**6 (3%)**

A total of 153 patients who underwent total hip replacement were successfully contacted twice to assess the adapted Goodman scale. The median age was 67 years (range, 23–75 years; interquartile range, 58–75 years), and 106 patients were women (70.7%) (Table 1). No major complications were observed at the end of follow-up.

**Fig. 2:**
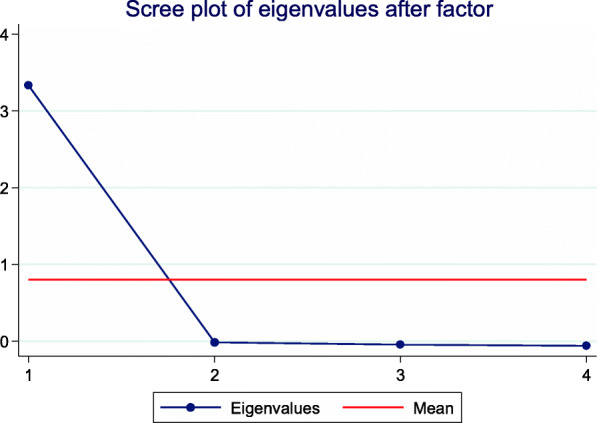
Screen plot of eigenvalues after factor analysis. Only 1 factor reached an eigenvalue above 1. This factor explains 90% of the answers

The Cronbach’s alpha was acceptable, reaching a coefficient of 0.95 (95% confidence interval, 0.82–1). No statistical difference (*t* test, *p* = 0.55) was found with the original 0.92 alpha coefficient reported by Goodman et al. [6]. The factorial analysis showed great internal validity as it isolated only 1 factor with an eigenvalue of 3.33 (Fig. 2). This shows that 1 answer accounted for 90% of the score, reinforcing that this cultural adaptation achieved a high level of internal consistency.

Test re-test concordance was achieved among the 3 tests. The ICC was 0.87 (95% confidence interval, 0.84–0.91), CCC was 0.87 (95% confidence interval, 0.84–0.91), and the Bradley-Blackwood test reached a probability of 0.1, meaning that all 3 concordance assessments were optimal [16].

The Spearman correlation showed a moderate correlation between the OHS and the Spanish-adapted Goodman scale. In the first round, rho was − 0.64 for patient satisfaction and 0.66 for change in quality of life. Meanwhile, in the second round, the rho estimated was − 0.63 in patient satisfaction and 0.61 to improve the quality of life.

## Discussion

This study describes a reliable and consistent Spanish adaptation of the Goodman scale, which evaluated patient satisfaction and quality of life after THA in Spanish speakers. Before using a recognized instrument to assess patients’ reported outcomes, it is imperative to perform a systematic translation and cultural adaptation [17].

Currently, PROMs are the most commonly used to compare surgical procedures in orthopaedic literature [18, 19]. PROMs have recently been used in national registries and could be helpful in decision-making in total joint arthroplasty and as an early indicator in implant failure [20, 21]. However, it is crucial to limit their use to evaluate aspects for which the scale was constructed for and to interpret their results according to their psychometric qualities [22, 23]. Moreover, Gagnier et al. [2] reported that no single PROM can evaluate all dimensions and have the required psychometric properties. This highlights the contribution of this work, adding feasibility and ease of management with proven psychometric properties to evaluate patient satisfaction and quality of life. This dimension was not addressed in the PROMs available in Spanish. Other characteristics of this scale are that it is available for free and can be self-administered on paper, by phone, or e-mail.

Patient satisfaction is a goal that every hip surgeon yearns for his patient and accurately measuring it is a keystone. Despite its relevance, a lack of instruments to assess this critical dimension has been reported [24]. Goodman et al. [8] reported in 2020 this promising tool, which we consider is important to be available in Spanish, given the number of Spanish speakers worldwide [9]. We did not find drawbacks during translation and cross-cultural adaptation; therefore, we encourage other surgeons interested in adapting this scale to their language.

Additionally, we report 86% of scored 75 points in the satisfaction dimension, and 88% said that their quality of life had a “great improvement” or “more than I ever dreamed possible,” which is consistent with the great experience reported by patients after THA [25] and to the original report of Goodman et al. [8].

A concern in this study is that no strong validation has been reported for OHS. The correlation with the OHS was good, as expected, because the OHS aimed to evaluate pain and function, which is not necessarily related to patient satisfaction or the change in quality of life that the Goodman scale seeks to assess. Moreover, Yeo et al. [26] recently reported that OHS is not a good PROM for evaluating satisfaction after THA. Nevertheless, the OHS is widely used, available in Spanish, and its psychometric properties have been rigorously tested [11, 27]. Therefore, in the absence of another Spanish test that addresses patient satisfaction, it seemed appropriate, and obtaining a reasonably good correlation should not question the validity of the score.

A limitation of this study is that a high percentage of patients could not complete the 2 rounds of answers. An explanation is that a portion of our population has digital illiteracy, limiting their accountability to answer e-mails. Another limitation is that no clinical outcomes were measured, such as range of motion. This study was carried out during the corona virus disease-2019 outbreak, making it unpropped to cite them in person; nevertheless, the original article did not measure clinical parameters. Additionally, this study has only a minimum of 1-year follow-up, but this should not affect the psychometric properties, even though an extensive follow-up could be necessary for future studies.

## Conclusions

The Spanish version of the Goodman questionnaire in THA is a reliable, consistent, and feasible scale to evaluate patient satisfaction and improve the quality of life of Spanish speakers. The excellent psychometric properties reported in this study are comparable to those of the original English version.

## Supplementary Information

**Additional file 1.** Adaptación cultural al español de la encuesta de Goodman.pdf. Cuestionario de Goodman en español. Spanish version of the Goodman questionnaire and instructions for the patients to complete it.

## Data Availability

All data generated or analyzed during this study are included in this published article and its supplementary information files.

## Abbreviations

PROMs: Patient-reported outcome measures
THA: Total hip arthroplasty
OHS: Oxford hip scales
ICC: Intraclass correlation coefficient
CCC: Lin's concordance correlation coefficient
