# Supplementary material for: Validation of the Spanish version of the Goodman score in total hip arthroplasty
Source: J Orthop Surg Res. 2021 Aug 20;16:517. doi: 10.1186/s13018-021-02653-6 (PMC8377152; doi:10.1186/s13018-021-02653-6)
Supplement: Supplementary file 1 — Additional file 1. Adaptación cultural al español de la encuesta de Goodman.pdf. Cuestionario de Goodman en español. Spanish version of the Goodman questionnaire and instructions for the patients to complete it. [file 13018_2021_2653_MOESM1_ESM.pdf]

Cuestionario de Goodman en español

Estimado paciente, nos gustaría saber su opinión sobre cuan satisfecho está luego de la Cirugía de su Prótesis de cadera.

Para esto, le pedimos que complete este breve cuestionario de 5 preguntas, marcando con una X el casillero que más represente su satisfacción según cada pregunta.

1. ¿Qué tan satisfecho está con los resultados de su cirugía de cadera para aliviar el dolor?
- ☐ Muy satisfecho
- ☐ De alguna manera satisfecho
- ☐ Ni satisfecho ni insatisfecho
- ☐ De alguna manera insatisfecho
- ☐ Muy insatisfecho

2. ¿Qué tan satisfecho está con los resultados de su cirugía de cadera para mejorar tu habilidad para hacer tareas domésticas o trabajar en el jardín?
- ☐ Muy satisfecho
- ☐ De alguna manera satisfecho
- ☐ Ni satisfecho ni insatisfecho
- ☐ De alguna manera insatisfecho
- ☐ Muy insatisfecho

3. ¿Qué tan satisfecho está con los resultados de su cirugía de cadera para mejorar sus habilidades en actividades recreativas?
- ☐ Muy satisfecho
- ☐ De alguna manera satisfecho
- ☐ Ni satisfecho ni insatisfecho
- ☐ De alguna manera insatisfecho
- ☐ Muy insatisfecho

4. En general ¿Qué tan satisfecho está con los resultados de su cirugía de cadera?
- ☐ Muy satisfecho
- ☐ De alguna manera satisfecho
- ☐ Ni satisfecho ni insatisfecho
- ☐ De alguna manera insatisfecho
- ☐ Muy insatisfecho

5. ¿Cuánto mejoró su calidad de vida después de la cirugía de cadera?
- ☐ Más de lo que alguna vez soñé
- ☐ Gran mejoría
- ☐ Mejoría moderada
- ☐ Una pequeña mejoría

- ☐ Ninguna mejoría en absoluto
- ☐ Mi calidad de vida es peor.
